# Calcar fracture gapping: a reliable predictor of anteromedial cortical support failure after cephalomedullary nailing for pertrochanteric femur fractures

**DOI:** 10.1186/s12891-021-04873-7

**Published:** 2022-02-24

**Authors:** Hui Song, Shi-Min Chang, Sun-Jun Hu, Shou-Chao Du, Wen-Feng Xiong

**Affiliations:** Department of Orthopaedic Surgery, Yangpu Hospital, School of Medicine, Tongji University, Shanghai, 200090 China

**Keywords:** Pertrochanteric fracture, Calcar fracture gapping, Cephalomedullary nailing, Anteromedial cortical support, Fracture reduction quality

## Abstract

**Background:**

Maintaining anteromedial cortical support is essential for controlling sliding and decreasing postoperative implant-related complications. However, adequate fracture reduction with cortical support in immediate postoperative fluoroscopy is not invariable in postoperative follow-ups. This study was conducted to investigate the risk factors leading to anteromedial cortical support failure in follow up for pertrochanteric femur fractures treated with cephalomedullary nails.

**Methods:**

This retrospective study enrolled 159 patients with pertrochanteric fractures (AO/OTA- 31A1 and 31A2) that fixed with cephalomedullary nails. All patients were evaluated as adequate fracture reduction in immediate postoperative fluoroscopy before leaving the operation theater. The patients were separated into two groups based on the condition of the anteromedial cortex in the postoperative 3D CT with full-range observation: those with calcar support maintained in Group 1 and those with calcar support lost in Group 2. Demographic information, fracture classification, TAD (tip-apex distance), Cal-TAD, Parker ratio, NSA (neck-shaft angle), reduction quality score, and calcar fracture gapping were collected and compared. Logistic regression analysis was conducted to explore the risk factors leading to anteromedial cortex change.

**Results:**

Anteromedial cortical support failure was noted in 46 cases (29%). There was no significant difference between the two groups concerning age, sex, side injury, TAD, Cal-TAD, Parker ratio, or NSA. There was a significant difference in the AO/OTA fracture classification in univariate analysis but no difference in the multivariable analysis. The reduction quality score, calcar fracture gapping in the AP (anteroposterior), and lateral views were significantly associated with anteromedial cortical support failure in follow-up after cephalomedullary nailing in the multivariable analysis. The threshold value of calcar fracture gapping for the risk of loss was 4.2 mm in the AP and 3.8 mm in the lateral fluoroscopies. Mechanical complications (lateral sliding and varus) were frequently observed in the negative anteromedial cortical support group.

**Conclusions:**

Good reduction quality was a protective factor, and larger calcar fracture gapping in the AP and lateral views were risk factors leading to the postoperative loss of anteromedial cortical support. Therefore, we should pay close attention to fracture reduction and minimize the calcar fracture gap during surgery.

## Background

For pertrochanteric femur fractures, static rigid fixation leads to more non-union and fixation failure; dynamic limited sliding is recommended for ideal fracture union [1–3]. Recently, cephalomedullary nails have been favorable owing to their biomechanical advantages and minimally invasive surgery [4–7]. It is worth noting that helical blade/lag screw cut-out occasionally occurs with an incidence rate of 13 to 15% [8, 9]. Related studies have proven that maintaining anteromedial cortical support is essential for controlling sliding and decreasing postoperative implant-related complications [10–12]. However, the verified anteromedial cortical support in immediate postoperative fluoroscopy or radiography taken before the patient leaves the operation theatre does not always remain invariable [13–16]. Previous research found a 20% reduction loss rate of anteromedial cortical support in postoperative follow-ups [13, 17].

This study was conducted to explore the possible risk factors leading to postoperative changes in anteromedial cortical support. We hypothesized that unstable fracture type, poor fracture reduction quality, and larger residual calcar fracture gapping after intramedullary nail fixation would result in a higher failure rate of the anteromedial cortex apposition.

## Methods

### Patient data and inclusion criteria

After Ethics Committee approval and written informed consent obtained from all enrolled patients, patients diagnosed with 31A1 and 31A2 pertrochanteric fractures treated with PFNA-II (proximal femoral nail anti-rotation) between January 2017 and June 2020 were retrospectively analyzed. All the methods were carried out following the guidelines and regulations. The inclusion criteria were as follows: age ≥ 60 years old; isolated fracture occurred less than 2 weeks prior; complete imaging data including preoperative radiography, intraoperative fluoroscopy, CT scanning, and 3D reconstruction took 1 week after the operation; and radiographic follow-ups for at least 6 months. The exclusion criteria included pathological fractures and immediate postoperative fluoroscopic images showing negative cortical relations in either AP, lateral, or 30-degree oblique views, before the patient leaving the operative theatre.

The 3D CT reconstruction images were considered the standard to judge the anteromedial cortical support, as they provided 360-degree full range views of the cortex [13]. According to the postoperative 3D CT scanning, patients were divided into two groups: Group 1, maintenance of calcar support as shown with positive or anatomic anteromedial cortical relation, and Group 2, loss of calcar support as shown with negative cortical relation.

The clinical data comparison included sex, age, side involved, and fracture classification. The technical data included TAD, Cal-TAD, Parker ratio, NSA, fracture reduction quality score, and residual calcar fracture gapping. The fracture classification was based on the AO/OTA-2007 version [18]. This research classified pertrochanteric fractures into only two subgroups, A1 and A2, to reduce the discrepancy between the sub-classifications.

### Surgical procedure

The patients were placed in the supine position on a fracture traction table. With the guidance of intraoperative fluoroscopy, routinely closed reduction maneuvers were performed to obtain fracture alignment. Negative cortical positional relationships before nailing were not allowed in either AP or lateral projections. However, if the fracture reduction redisplaced after nailing, we accepted it, and no further manipulation was attempted, especially in older and frail patients.

### Postoperative management

Postoperative standing and walking were essential for secondary stability. The rehabilitation process was individually tailored according to the patient’s physical capability and willingness. With good physical strength, early weight-bearing standing and walking were encouraged; otherwise, bed rest was recommended for 1 month, with no sitting or turning restrictions.

### Parameter measurement

**Fig. 1:**
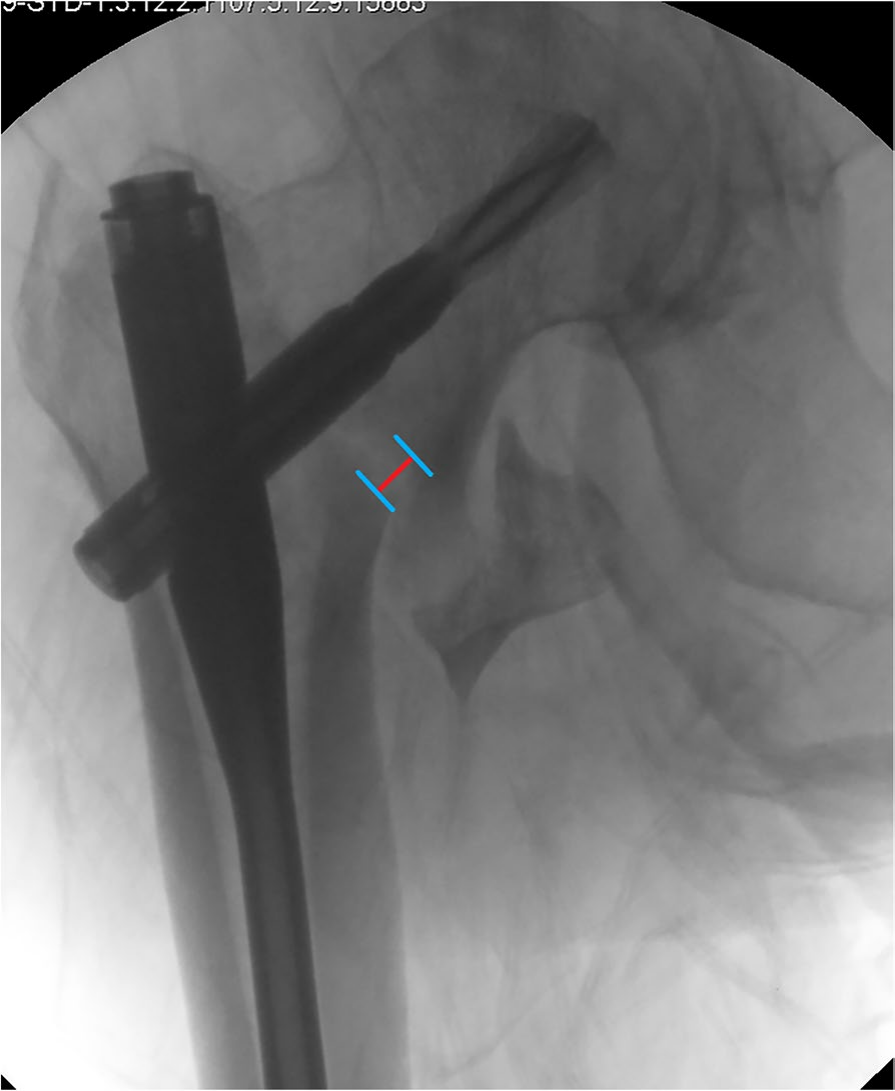
Calcar residual fracture gapping measurement at the medial basicervical in the AP view

**Fig. 2:**
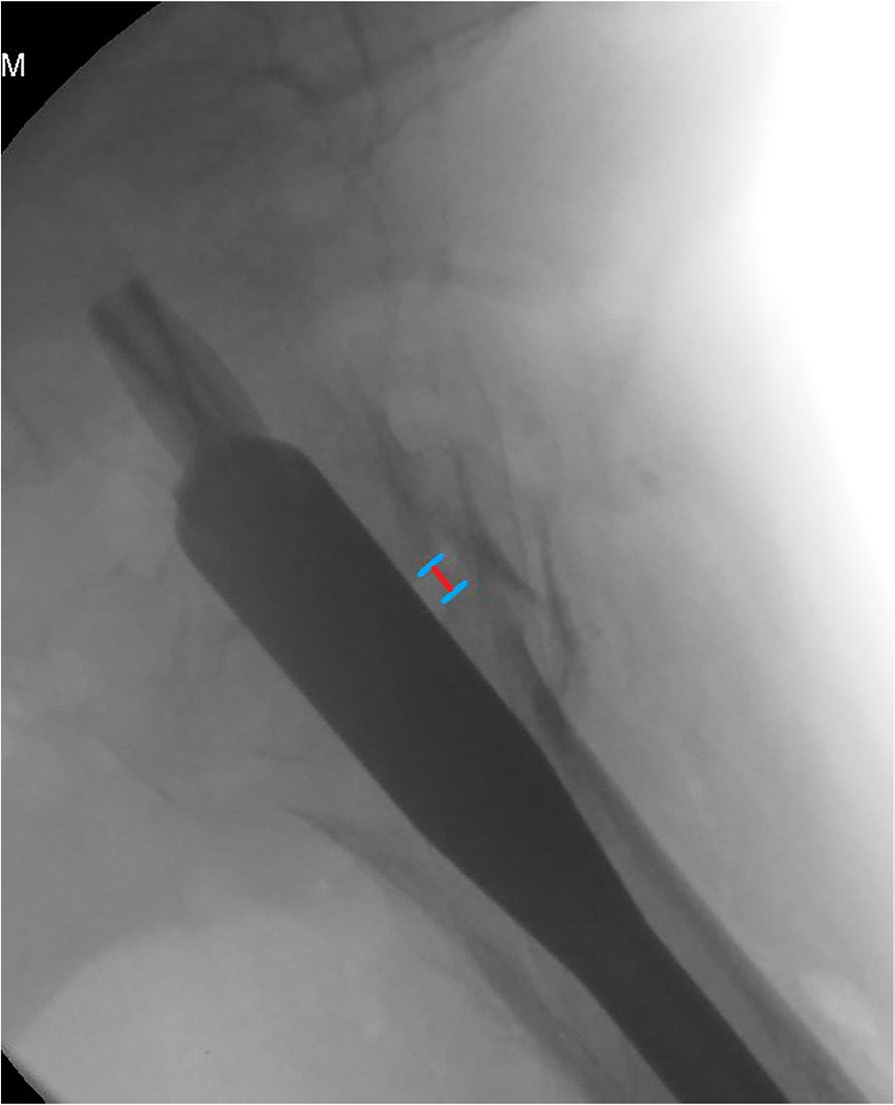
Anterior fracture gapping in the lateral view, defined parallel to the sliding direction of the head-neck fragment

Clinical and technical data were collected and measured by two independent researchers. One was a fellowship-trained orthopaedic surgeon, and the other was an orthopaedic resident. The mean value of the data calculated by the two observers was used for statistical analysis. A senior professor joined the discussion if any disagreement occurred. The intra-class correlation coefficient (ICC) was calculated to judge the intra-observer reliability and was determined to be good (> 0.800). Standard AP, lateral (axial), and anteromedial oblique fluoroscopic views were acquired in the operating room. Immediate postoperative radiographic images were used to calculate technical data such as TAD, Cal-TAD, Parker ratio in the AP and lateral views, NSA, and calcar fracture gapping in the AP and lateral views. The technical data were calculated by using Photoshop CC 2018 (Adobe) software with the pixel ruler technique. The measurement of calcar fracture gapping in the AP view was calculated according to previously described studies [19, 20] (Fig. 1). The lateral view was measured parallel to the sliding direction of the head-neck fragment (Fig. 2). The TAD, Cal-TAD, Parker ratio, NSA, and lateral sliding distance were calculated as previously described [21–23]. The assessment of the fracture reduction quality was evaluated according to the four-point criteria proposed by Prof. Chang [11].

### Outcomes

The primary outcome was defined as the degree of fracture gapping in the AP/lateral views in the immediate postoperative fluoroscopies, which predict loss of anteromedial calcar support in postoperative follow up. The follow-up time was set to be more than 6 months, as the fractures could either have union or fixation failure occurred [20, 24]. Mechanical complications were defined as varus displacement (NSA reduced > 10° postoperatively) [25] and/or excessive lateral sliding of the helical blade (lateral sliding ≥10 mm postoperatively) [26].

### Statistical analysis

SPSS 22.0 software was used for the statistical analysis (SPSS Inc., Chicago, IL, USA). Continuous data are presented as the mean and SD (standard deviation). Categorical data are presented as counts and percentages. For the frequencies, the *x*^2^ or Fisher exact test was conducted for univariate analysis. The t-test or the Mann–Whitney U test was performed for univariate analysis to compare the means. Variables with a *p*-value less than 0.10 were further included in the multivariable analysis. The receiver operating characteristic (ROC) curve was used to estimate the threshold value of calcar fracture gapping for predicting anteromedial cortex alterations. A *p*-value less than 0.05 was defined as statistically significant.

## Results

**Fig. 3:**
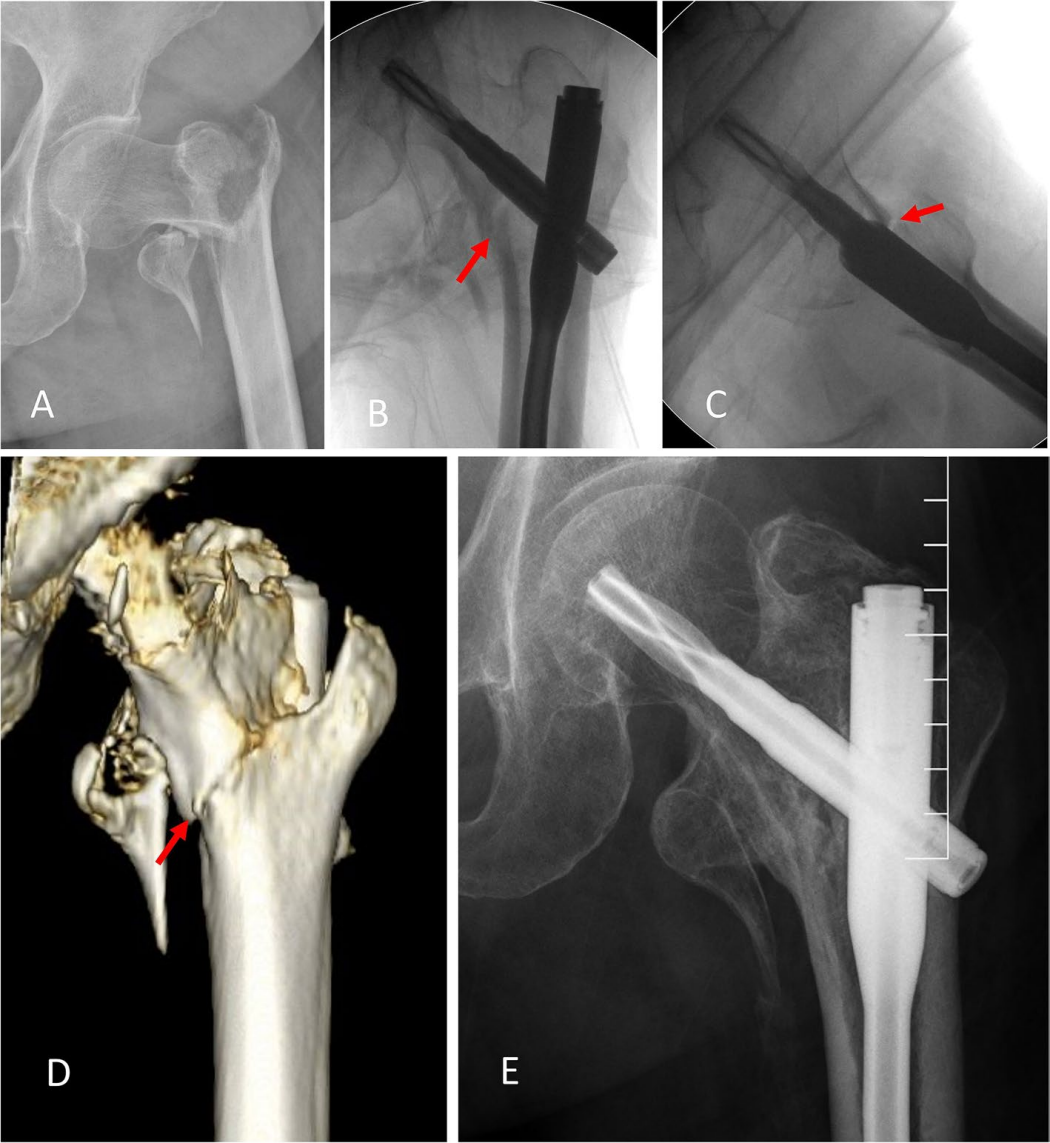
An illustrative case of maintenance-of-calcar support in postoperative 3D-CT follow-up. **A**: An 86-year-old female diagnosed with 31A2.3 trochanteric fracture. **B**: Immediate AP fluoroscopy after operation showed positive relation of the two medical cortices; the arrow indicated a close contact of the inferior medial calcar. **C**: Immediate lateral fluoroscopy after operation showed neutral relation of the two anterior cortices; the arrow indicated minimal gap at the anterior cortex. **D**: Postoperative 3D CT image showed true positive anteromedial cortical support at the inferior corner. **E**: In postoperative 26 months follow-up, the fracture healed with minimal lateral sliding of the helical blade

**Fig. 4:**
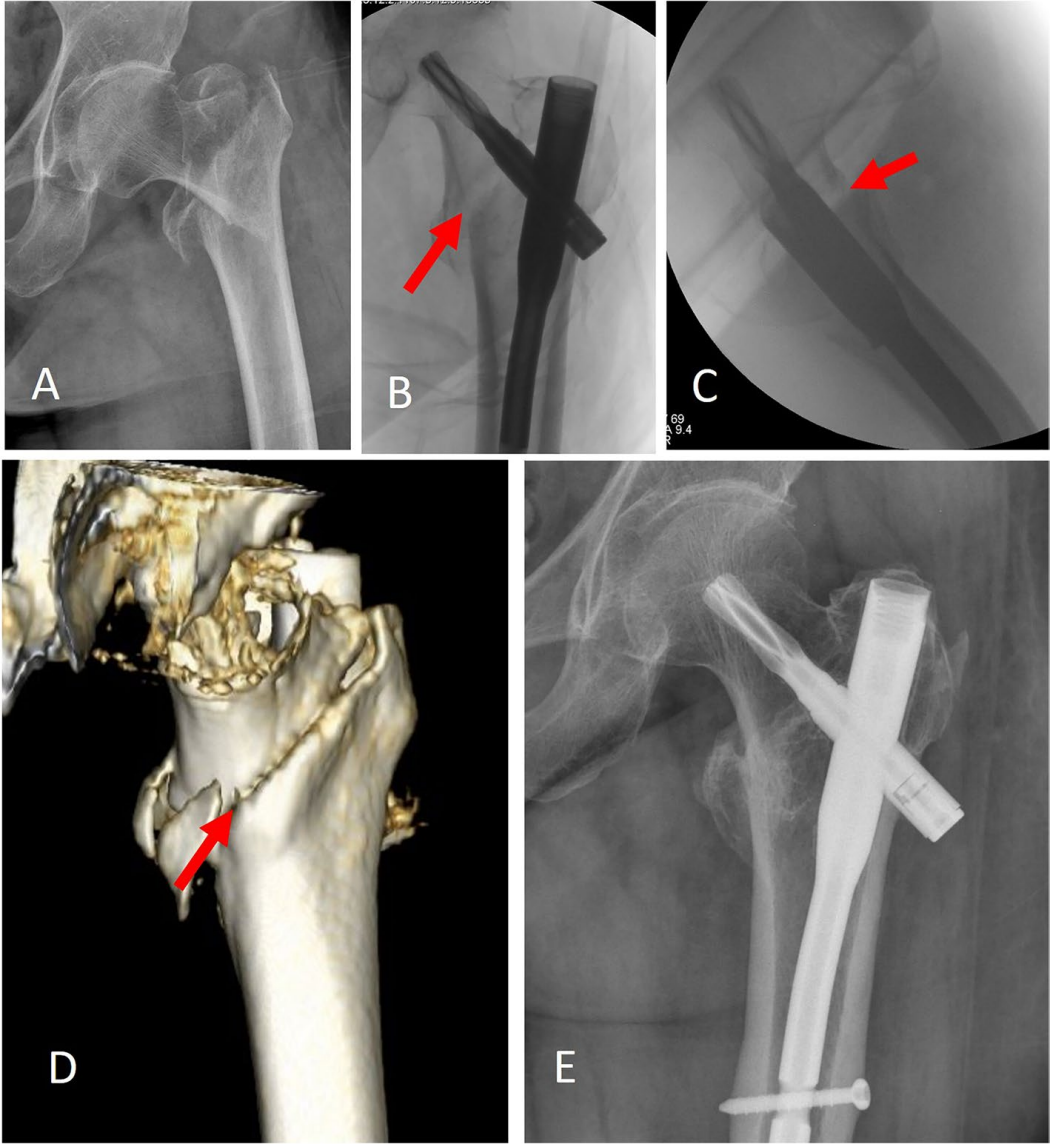
An illustrative case of loss-of-calcar support in postoperative 3D-CT follow-up. **A**: A 77-year-old female, diagnosed with 31A2.2 trochanteric fracture. **B**: Immediate AP fluoroscopy after operation, the arrow showed a large calcar fracture gap of the medial cortex (4.83 mm). **C**: Immediate lateral fluoroscopy after operation, the arrow showed a large calcar fracture gap of the anterior cortex (3.61 mm). **D**: Postoperative 3D CT image showed negative anteromedial cortical support. The arrow indicated posterior sagging of the head-neck fragment. **E**: In postoperative 14 months follow-up, the AP radiograph showed an apparent lateral sliding of the helical blade (10.5 mm)

In total, 159 cases were included in this study. Anteromedial cortical support failure was noted in 46 patients (29%). The demographic information and technical data comparison between the two groups are shown in Table 1. There was no significant difference in age, sex, side of injury, TAD, Cal-TAD, Parker ratio, or NSA. There was a considerable difference concerning fracture classification, reduction quality score, and calcar fracture gapping in AP and lateral views. However, only good reduction quality score was an independent protective factor, and calcar fracture gapping in AP, and lateral views were independent risk factors in the multivariable analysis (Table 2). Mechanical complications are shown in Table 3. The loss-of-calcar support group suffered inferior mechanical complications compared with the maintenance-of-calcar support group (Fig. 3 and Fig. 4).

**Table 1:** Demographic and clinical data comparison between Group 1 and Group 2

Characteristic	Group 1 (*n* = 113)	Group 2 (*n* = 46)	*p* value
Age (years)	82.0 ± 9.2	84.4 ± 7.0	0.116
Sex (%)			0.114
Male	39 (34.5)	10 (21.7)	
Female	74 (65.5)	36 (78.3)	
Side (%)			0.757
Left	51 (45.1)	22 (47.8)	
Right	62 (54.9)	24 (52.2)	
AO/OTA classification (%)			0.005
A1	52 (46.0)	10 (21.7)	
A2	61 (54.0)	36 (78.3)	
Reduction quality score; Good/Acceptable (%)	104/9 (92.0/8.0)	11/35 (23.9/76.1)	< 0.001
NSA (degree)	130.8 ± 6.0	131.0 ± 5.3	0.810
TAD (mm)	24.00 ± 7.19	23.68 ± 6.97	0.794
Cal-TAD (mm)	24.06 ± 5.87	25.00 ± 6.91	0.387
Calcar fracture gapping in AP view (mm)	2.36 ± 1.57	7.09 ± 2.70	< 0.001
Calcar fracture gapping in lateral view (mm)	2.22 ± 1.61	5.89 ± 3.27	< 0.001
Parker ratio in AP view (%)	41.91 ± 7.84	43.32 ± 8.46	0.315
Parker ratio in Lat view (%)	47.12 ± 8.74	44.72 ± 8.32	0.114

**Table 2:** multivariable analysis to detect possible risk factors for losing anteromedial cortex-to-cortex support

Characteristic	OR	CI (95%)	*p* value
AO classification			
31.A1	0.749	0.160–3.506	0.713
31.A2	1.0	reference	
Reduction quality			
Good	0.097	0.022–0.430	0.002
Acceptable	1.0	reference	
Calcar fracture gapping in AP view (mm)	2.022	1.456–2.808	< 0.001
Calcar fracture gapping in lateral view (mm)	1.437	1.060–1.947	0.019

**Table 3:** Mechanical complications

Mechanical complications	Group 1 No. of cases (%)	Group 2 No. of cases (%)	*p* value
Varus displacement (> 10°)	3 (2.7)	10 (21.7)	< 0.001
Excessive lateral sliding (≥10 mm)	2 (1.8)	8 (17.4)	0.001

**Fig. 5:**
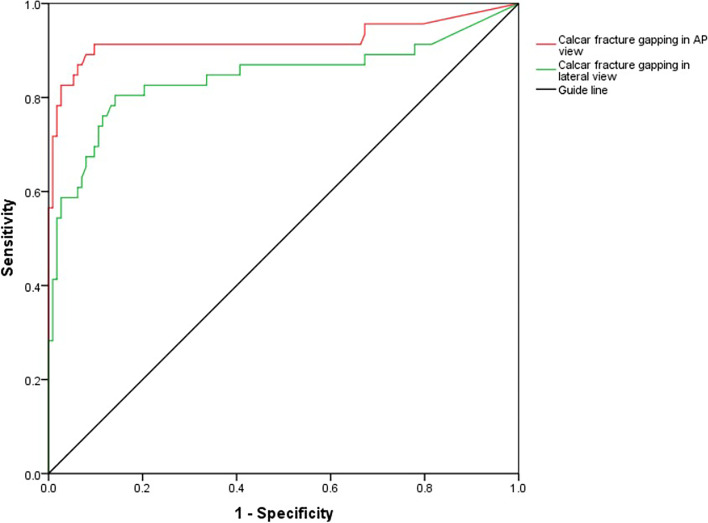
A graph demonstrating the ROC curve by plotting the sensitivity and the 1-specificity. For the calcar fracture gapping assessment, the recommended cut-off point for balancing sensitivity and specificity was 4.2 mm with 91.3% sensitivity and 90.3% specificity in the AP view and 3.8 mm with 80.4% sensitivity and 85.8% specificity in the lateral view. The area under the curve (AUC) was 0.922 for the AP view and 0.843 for the lateral view

According to the ROC curve threshold value analysis for postoperative anteromedial cortical support failure, the best cut-off point of calcar fracture gapping for balancing sensitivity and specificity was 4.2 mm in the AP view and 3.8 mm in the lateral view (Fig. 5).

## Discussion

This retrospective study investigated the risk factors leading to anteromedial cortical support failure in follow-ups for pertrochanteric fractures treated with cephalomedullary nails. The reduction quality and residual calcar fracture gapping between the head-neck and femoral shaft fragments in the AP and lateral views were reliable predictors of anteromedial cortical support alterations. These findings further developed the concept of cortical support theory with quantification.

### Anteromedial cortical support reduction

Anteromedial cortical support reduction was introduced by Chang et al. [11] in 2015 for pertrochanteric fractures. Anteromedial cortical support reduction is a non-anatomic buttress reduction that allows limited sliding along the lag screw/helical blade axis and achieves secondary stability, providing mechanical stability to share loads from the implant and biological environment for fracture healing [1, 13, 17]. In contrast, the mean loss of the femoral neck-shaft angle, neck shortening, varus deformity, healing complications, and implant failure in the negative medial cortical support group was significantly higher than positive/neutral medial cortical support groups [10, 12, 27]. Similarly, our research revealed that mechanical complications (including varus displacement and excessive lateral migration) were frequently observed in the negative anteromedial cortex support group.

### Postoperative alteration of anteromedial cortical support reduction

In 2018, Chang et al. [13] revealed the discrepancy of anteromedial cortical support reduction in pertrochanteric fractures between immediate postoperative fluoroscopy and postoperative 3D CT reconstructions. Acceptable anteromedial cortical contact in both AP and lateral views (positive or neutral) endured an approximately 20% loss rate of cortical support postoperatively. Furthermore, Chen et al. [17] added a 30-degree oblique view (displaying the anteromedial inferior corner cortices) to estimate anteromedial cortex contact status. The change rate still reached up to 10%. However, the possible risk factors predicting the change in the final cortical contact are still out of reach. Therefore, this research preliminarily revealed that fracture reduction and calcar fracture gapping played an essential role in inducing the alteration of anteromedial cortical support.

### Importance of fracture reduction quality

In 1980, Prof. Kaufer [28] proposed five factors influencing the treatment outcomes of trochanteric fractures: bone quality, fracture morphology, implant choice, implant placement, and fracture reduction quality. The first two factors belong to fracture characteristics and are non-modifiable. The last three factors are modifiable and could be controlled by the orthopaedist. Among the five factors, fracture reduction is the first manipulation during operation and is regarded as paramount compared with other factors [29].

In 1995, Baumgartner et al. [24] proposed the criteria for fracture reduction quality, including both the alignment and displacement degree of the main fragments. The fracture reduction quality was rated as good, acceptable, and poor. Based on the alignment and medial/anterior cortex contact status, Chang and colleagues further proposed [11] a new fracture reduction criterion for trochanteric fractures, which is more reliable in indicating postoperative mechanical complications than the Baumgaertner reduction system [12]. Studies have revealed that poor reduction quality is an independent risk factor for implant failure and inferior outcomes [19, 30]. Similarly, this study found that good reduction quality was an independent protective predictor of anteromedial cortical support alteration in pertrochanteric fractures treated with intramedullary nails (OR, 0.097; 95% CI, 0.022–0.430; *p* = 0.002). Our result was consistent with previous studies concerning the importance of fracture reduction quality. Therefore, we should emphasize the importance of reduction quality before the intramedullary nailing process.

### Calcar fracture gapping

During the surgery, anteromedial calcar fracture gapping was frequent. Zhang et al. [31, 32] reported that an inferior medial gap (mean and standard deviation, 9.2 ± 4.6 mm) appears during intramedullary nailing in basicervical trochanteric fractures, called the reverse wedge effect. In addition, inferior placement of the guidewire is beneficial for acquiring an ideal Cal-TAD. However, when the spiral blade is hammered into the head and neck bone, it easily impinges on the bone block due to the narrow local space and promotes separation of the bone block, which is called the impingement effect [33]. It is worth noting that calcar fracture gapping is also a subset criterion for fracture reduction quality. Baumgaertner reduction quality criteria considered the displacement of fragments in the AP or lateral view of more than 4 mm as unacceptable criteria [24, 34].

Existing larger calcar fracture gapping is associated with a poor prognosis. Parry et al. [20] revealed that calcar gapping and fracture classification was related to over sliding of lag screws leading to revision surgeries, which is consistent with our results. Ciufo et al. [19] also revealed that basicervical gapping (defined as cortical diastasis > 3 mm) and mal-reduction were risk factors for cutting out in trochanteric fractures treated by cephalomedullary nails. Lobo-Escolar et al. [35] demonstrated that diastasis of fragments > 3 mm postoperatively was significantly more common in the cut-out group treated with femoral intramedullary nailing. In this research, we discovered that calcar fracture gapping in the AP (OR, 2.022; 95% CI, 1.456–2.808; *p* < 0.001) and lateral (OR, 1.437; 95% CI, 1.060–1.947; *p* = 0.019) views were risk factors leading to the negative transformation of anteromedial cortical support.

### Mechanisms lead to the change

Limited fracture gapping is beneficial for subsequent fragment sliding and secondary stability to achieve medial cortex-to-cortex contact. However, the cortex-to-cortex buttress will not be realised if the space is larger than one cortical thickness. Due to the biomechanical characteristics of the hip joint, the head-neck fragment obliquely slides in the inferior lateral direction until it contacts the intramedullary nail, which gains implant support. Therefore, the existing larger calcar gap might lead to the loss of anteromedial cortical support during the sliding process [1]. In addition to calcar fracture gapping, other factors might also interfere with sliding and change anteromedial cortical support. Possible factors include the capacity to activate head-neck sliding, the orientation of sliding, external rotation of the femoral shaft, and rotation during sliding.

### Study limitations

The limits of this research were the retrospective analysis and the number of cases enrolled. The intraoperative fluoroscopic images were low in resolution, which possibly led to an error during the measurement. However, intraoperative CT was not always available in the majority of operating theatres. The pixel ratio calculation method was beneficial for decreasing the error. Osteoporosis severity was not analysed in this research, as we considered that patients over 60 years old who suffered from low-energy fractures could be diagnosed with severe osteoporosis.

## Conclusions

For pertrochanteric femur fractures fixed with cephalomedullary nails, poor reduction quality and larger calcar fracture gapping in the AP and lateral views were risk factors leading to changes in anteromedial cortical support. Mechanical complications more frequently occurred in the group that lost anteromedial cortex support. Therefore, before inserting the nail, good reduction quality should be realized, and calcar fracture gapping in the AP and lateral views should be controlled to be less than 4 mm. Close contact of the fragments is beneficial for decreasing the sliding of the head and neck fragment and ensuring anteromedial cortical support at the anteromedial corner.

## Data Availability

The datasets used and analyzed during the current study are available from the corresponding author on reasonable request if requested (please contact shiminchang11@aliyun.com).

## Abbreviations

3D-CT: Three dimensional computed tomography
TAD: Tip apex distance
Cal-TAD: Calcar referenced tip-apex distance
NSA: Neck shaft angle
AP: Anteroposterior
ICC: Intra-class correlation coefficient
ROC: Receiver Operating Characteristics
AUC: Area under the curve
